# The Protective Effect of Adenoidectomy on Pediatric Tympanostomy Tube Re-Insertions: A Population-Based Birth Cohort Study

**DOI:** 10.1371/journal.pone.0101175

**Published:** 2014-07-01

**Authors:** Mao-Che Wang, Ying-Piao Wang, Chia-Huei Chu, Tzong-Yang Tu, An-Suey Shiao, Pesus Chou

**Affiliations:** 1 Department of Otolaryngology Head Neck Surgery, Taipei Veterans General Hospital, Taipei, Taiwan and School of Medicine, National Yang-Ming University, Taipei, Taiwan; 2 Institute of Public Health and Community Medicine Research Center, National Yang-Ming University, Taipei, Taiwan; 3 Department of Otolaryngology Head Neck Surgery, Mackay Memorial Hospital, Taipei, Taiwan and Department of Audiology and Speech Language Pathology and School of Medicine, Mackay Medical College, New Taipei City, Taiwan; Fondazione IRCCS Ca' Granda Ospedale Maggiore Policlinico, Università degli Studi di Milano, Italy

## Abstract

**Objectives:**

Adenoidectomy in conjunction with tympanostomy tube insertion for treating pediatric otitis media with effusion and recurrent acute otitis media has been debated for decades. Practice differed surgeon from surgeon. This study used population-based data to determine the protective effect of adenoidectomy in preventing tympanostomy tube re-insertion and tried to provide more evidence based information for surgeons when they do decision making.

**Study Design:**

Retrospective birth cohort study.

**Methods:**

This study used the National Health Insurance Research Database for the period 2000–2009 in Taiwan. The tube reinsertion rate and time to tube re-insertion among children who received tympanostomy tubes with or without adenoidectomy were compared. Age stratification analysis was also done to explore the effects of age.

**Results:**

Adenoidectomy showed protective effects on preventing tube re-insertion compared to tympanostomy tubes alone in children who needed tubes for the first time (tube re-insertion rate 9% versus 5.1%, *p* = 0.002 and longer time to re-insertions, *p* = 0.01), especially those aged over 4 years when they had their first tube surgery. After controlling the effect of age, adenoidectomy reduced the rate of re-insertion by 40% compared to tympanostomy tubes alone (aHR: 0.60; 95% CI: 0.41–0.89). However, the protective effect of conjunction adenoidectomy was not obvious among children with a second tympanostomy tube insertion. Children who needed their first tube surgery at the age 2–4 years were most prone to have tube re-insertions, followed by the age group of 4–6 years.

**Conclusions:**

Adenoidectomy has protective effect in preventing tympanostomy tube re-insertions compared to tympanostomy tubes alone, especially for children older than 4 years old and who needed tubes for the first time. Nonetheless, clinicians should still weigh the pros and cons of the procedure for their pediatric patients.

## Introduction

Acute otitis media (AOM) and otitis media with effusion (OME) are very common otologic problems in children. The middle ear cavity is filled with infected fluid and the mucosa is inflamed. Ninety percent of children experience AOM and OME before school age, most often between 6 months and 4 years of age [1], [2]. Most OME resolve spontaneously within three months, but 30–40% may have recurrent OME and 5–10% of episodes may last for a year or longer [1], [3], [4]. Diagnosis of OME depends on history, including previous rhino-sinusitis or AOM, decreased hearing noted by the care giver, inattention at school, and aural fullness sensation as stated by the child. Physical examination is based mainly on pneumatic otoscopy, which is an inexpensive, accessible, and easily used diagnostic tool [3], [5]. Diagnosis may be confirmed by telescopy, pure tone audiometry, and tympanometry [6]. Management includes conservative treatment and surgical intervention. The American Academy of Otolaryngology Head and Neck Surgery (AAO-HNS) set the clinical practice guidelines for OME in 2004. Based on the self-limiting nature of most OME, clinicians should manage children who are not at risk by watchful waiting for three months from the date of effusion onset (if known) or from the date of diagnosis (if onset is unknown). If a child becomes a surgical candidate, tympanostomy tube insertion is the preferred initial procedure. Adenoidectomy should only be performed when there is nasal obstruction or chronic adenoiditis, or in repeated tympanostomy tube insertions. Tonsillectomy or myringotomy alone should not be used [5]. The AAO-HNS also set clinical practice guidelines for tympanostomy tubes in children in 2013, recommending that clinicians offer bilateral tympanostomy tubes to children with bilateral chronic OME (OME last for 3 months or longer), and recurrent AOM with middle ear effusion. The guideline also recommended that clinicians should not offer tympanostomy tubes to children with single episode of OME lasting less than 3 months, and recurrent AOM without middle ear effusion [7].

For children with tympanostomy tubes, 20–50% may require repeated tympanostomy tubes after their initial tubes extruded [8]–[10]. Adenoidectomy has been proved to be effective in preventing recurrence of OME, recurrent AOM, or the need for repeated tympanostomy tubes in many studies in the past 30 years [11]–[20], and only a few demonstrated contrary data [21]–[24]. Adenoidectomy may reduce repeated tympanostomy tubes by 50% [15]–[19]. Why is adenoidectomy effective in preventing pediatric middle ear infection? The adenoids are considered an important factor in pediatric middle ear infection since it may be a reservoir of pathogens [25], while its size effect may block the Eustachian tube orifice [26], [27]. Thus, it may play a role in middle ear inflammation or decreased ciliated mucosa [28]–[30]. However, it is not suggested as a regular procedure in treating chronic OME or recurrent AOM or in conjunction with primary tympanostomy tube insertions [5], [31], for the possible complications of general anesthesia and the procedure itself like bleeding, nasopharyngeal stenosis, and injury to the orifice of Eustachian tubes [32]–[34]. Although the AAO-HNS practice guidelines for OME suggested adenoidectomy only for children requiring repeated tympanostomy tubes [5], many surgeons performed adenoidectomy in conjunction with tympanostomy tubes insertion as the initial treatment for chronic OME or recurrent AOM in recent years after the release of AAO-HNS practice guidelines [16], [18], [19]. When to perform adenoidectomy for children with chronic OME remains a major debatable issue. Another controversial issue is the age at which adenoidectomy will be beneficial to children with chronic OME. Many studies show that adenoidectomy is only beneficial to children of certain age groups. In three studies, Gates et al. and Maw showed that adenoidectomy was beneficial in children with OME older than 4 years [11], [12], [14], and one most recent systemic review and metanalysis also concluded that adenoidectomy with primary tube insertion appears to provide a protective effect against repeated surgery in children older than 4 years [35], while Hammaren-Malmi et al. demonstrated that adenoidectomy did not reduce OME in children younger than 4 years old [21]. However, Coyte et al. found that adenoidectomy was beneficial to children older than 2 years old and that the benefits were more obvious among children older than 3 years old [15]. Thus, the results of these studies are not consistent. This population-based retrospective birth cohort study aimed to examine the protective effect of adenoidectomy for tube re-insertion using the National Health Insurance Research Database (NHIRD) in Taiwan. Specifically, this study examined the efficacy of adenoidectomy in conjunction with tympanostomy tube insertion for reducing the repeated tympanostomy tubes compared to tympanostomy tubes alone. We used Tympanostomy tube insertion as a surrogate for chronic OME and recurrent AOM because surgical procedures were usually for most serious and retractable cases. Besides, the reduction of tube insertion also means the reduction of the risk of general anesthesia and the procedure itself which were really burdens for both pediatric patients and their parents. The National Health Insurance (NHI) in Taiwan, established since 1995, has a nationwide coverage of more than 99% of legal residents. It is well known for its low fees and low reimbursement but high quality of service. All of the medical services and medication in Taiwan are paid for by NHI, which is also characterized by easy accessibility without a regulated referral system. Patients may go to any doctor or any hospital on their own will, with or without the referral of primary care physicians. All of the medical procedures and claims are recorded in the NHI database, which is the only buyer of medical service in Taiwan. The NHIRD is released for academic use yearly by the National Health Institute of Taiwan.

## Materials and Methods

The study was reviewed and approved by the Institutional Review Board of Taipei Veterans General Hospital. (IRB number: 2013-02-019B) No inform consent was given because this study analyzed government released secondary data. The identification of every individual in the database was censored. This ten-year study (2000–2009) used the Taiwan NHIRD, a population-based data on approximately 23 million people covered by the NHI. Every admission and outpatient visit record was included in this database without sampling. All children born in the year 2000 and 2001 who had tympanostomy tube insertion before the end of the study period (end of the year 2009) were included. They were divided into two groups based on whether or not adenoidectomy was done together with their first tympanostomy tube insertion. Data on these children was examined to determine if they received repeated tube insertions before the end of the study period.

**Figure 1:**
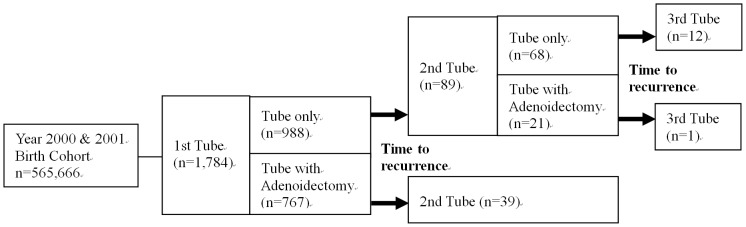
Study flow chart.

Those with repeated tube insertions without adenoidectomy on their first tympanostomy tube insertion were further divided into two groups based on whether or not adenoidectomy was done together with their second tympanostomy tube insertion. Data on these children was further examined to determine if they received a third tube insertion before the end of the study period (Fig. 1). The repeat tube insertion rate and time to repeated tubes were compared between children who received adenoidectomy with tympanostomy tubes and those who received tube insertion alone.

The study population was obtained by retrieving all of the patients with the procedure code for myringotomy with ventilation tube insertion under a microscope from 2000 to 2009 from the claims data of the NHIRD, with a birthday between January 1, 2000 and December 31, 2001. That is a population-based data without any sampling. As such, a population based year 2000 and 2001 birth cohort for tympanostomy tube insertion was obtained and followed-up to 8 or 9 years old. Children with cleft palate with diagnosis codes in International Classification of Disease, 9^th^ Revision (ICD-9) 749.00∼749.04 were excluded because they tended to have multiple tympanostomy tube insertions [36]–[38]. Adenoidectomy was also relatively contraindicated for children with cleft palate as it might lead to velo-pharyngeal incompetence [39]. Concurrent tympanostomy tube insertion and adenoidectomy was defined by identifying two procedure codes for myringotomy with ventilation tube insertion under a microscope, and for adenoidectomy on the same day in the claims data. Adenoidectomy done with tonsillectomy at the same time was also identified and was not included in this study.

The children were also stratified into four age groups in years in order to examine the effect of age (0≦age<2, 2≦age<4, 4≦age<6, and 6≦age<9). The rate of repeated tympanostomy tube insertion and time to recurrence were examined in each age group to explore the protective effect of adenoidectomy on tube reinsertion. The age group with highest risk of tube re-insertion was further determined. The rate of post-adenoidectomy bleeding was also explored.

### Statistical Analysis

The tube insertion rate between children with adenoidectomy and tympanostomy tubes and those with tympanostomy tubes alone in all age groups was compared using the Fisher's exact test. The time between the first tympanostomy tube insertion and repeated procedures in the study period was compared by log-rank test for failure time. The adjusted hazard ratio of recurrence between children with and those without adenoidectomy and among age groups was obtained by Cox proportional hazard model. The statistical results were obtained via the software SAS 9.1 (SAS Institute, Cary, NC, USA). Statistical significance was set at *p*<0.05. All values were expressed as mean ± standard deviation (SD).

## Results

According to the Taiwan National Statistics Report, there were 305,312 and 260,354 newborns in the year 2000 and 2001 respectively [40]. This study had a population-based birth cohort numbering 565,666 who were followed-up for 8 to 9 years. A total of 2221 children in the 2000 and 2001 birth cohorts had tympanostomy tube insertion before the age of 8 or 9 years. The cumulative incidence of tympanostomy tube insertion before 8 or 9 years of age was 0.393%. After excluding 437 children with cleft palate, and 29 children with adenotonsillectomy, 1755 were included in this study. Among them, 1627 cases had only one tube insertion before 8 or 9 years of age. There were 1065 males, or 60.7% of the total cases. Around 80% of children had their first tube surgery after 4 years of age. One hundred and eleven had two tubes insertions and 17 had more than two insertions. Additional adenoidectomy and age at tympanostomy tube insertions and adenoidectomy were shown in Table 1.

**Table 1:** **Descriptions of 2000–2001 birth cohort who had undergone tympanostomy tubes before 9 years of age.**

Characteristics	n	%
Total subjects	1755	100.0
Gender*		
Male	1065	60.7
Female	689	39.3
Age at 1st tube insertion*		
0–2 years	183	10.4
2–4 years	222	12.7
4–6 years	856	48.8
6–9 years	494	28.2
Number of chronic OME episodes		
1	1627	92.7
2	111	6.3
3	12	0.7
4+	5	0.3
Surgical operation		
Tube only	988	56.3
Tube + Adenoidectomy	767	43.7
Age at tube insertion†		
1st tube insertion	5.0	1.8
2nd tube insertion	5.9	1.5
3rd tube insertion	6.9	1.3
Age at adenoidectomy‡		
0–2 years	5	0.6
2–4 years	82	10.1
4–6 years	450	55.5
6–9 years	274	33.8

*One missing value.

† Shown by mean and standard deviation.

‡ Only those who had undergone adenoidectomy were included (n = 767).

**Figure 2:**
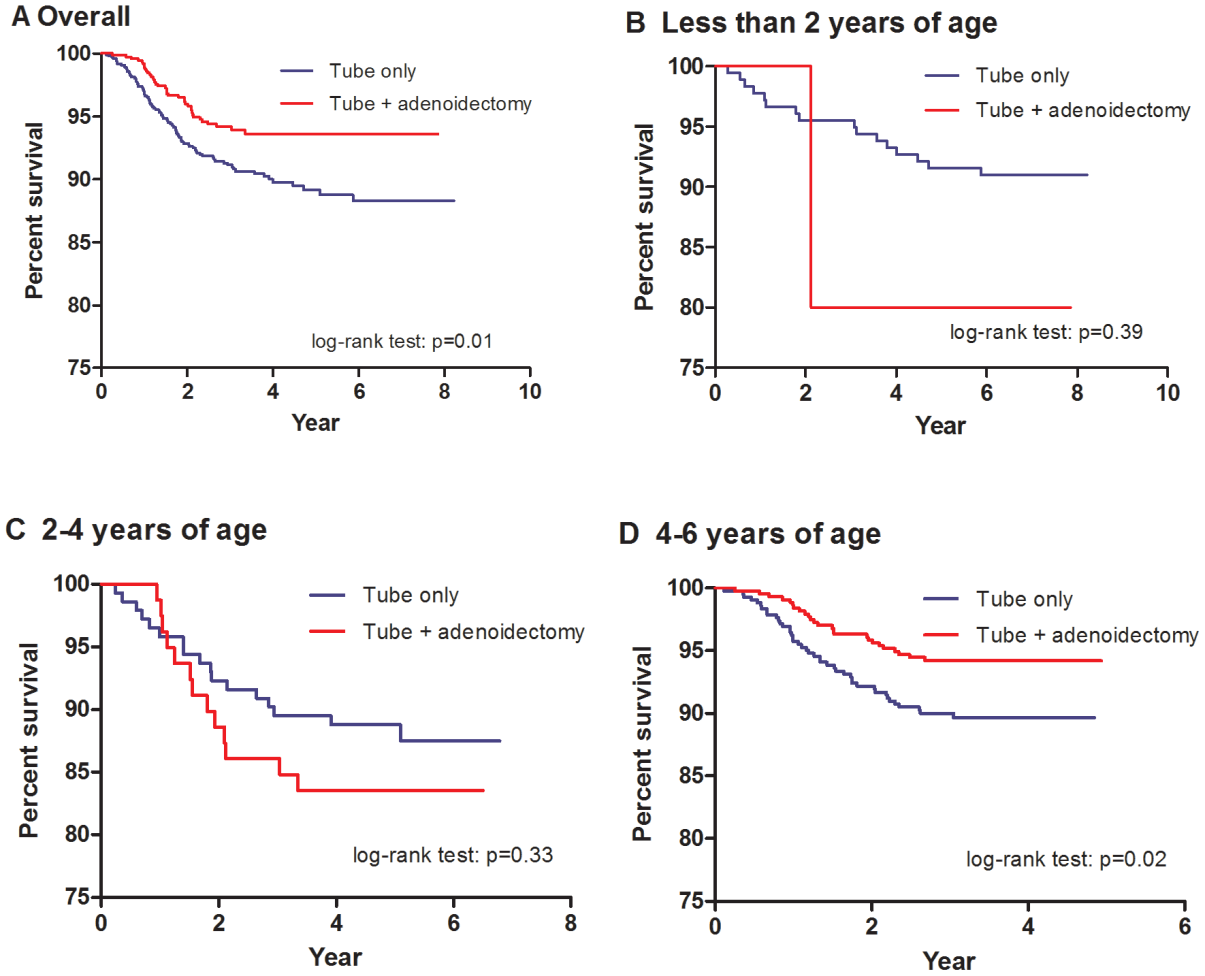
Survival curve of tube re-insertions. (A) Overall recurrence. (B) (C) and (D) Recurrence stratified by age.

Of the 1755 cases included, 767 had adenoidectomy on their first tympanostomy tube insertion. The other 988 children had tube insertion alone, although 89 of them needed repeated tube insertions. There were 21 who had adenoidectomy on their second tubes insertion while 68 had tube insertion only. The age of children received adenoidectomy was 5.5±1.3 (mean±SD) years old. Children who received both adenoidectomy and tympanostomy tubes on their first tubes insertion had a lower recurrence rate than those who had tubes alone (*p* = 0.002). They also had a longer time to re-insertions (*p* = 0.01) (Fig. 2). However, the protective effect of adenoidectomy on the second tube insertion was not observed in terms of re-insertion rate and in time to re-insertions (*p* = 0.29 and *p* = 0.22, respectively) (Table 2).

**Table 2:** **Tympanostomy tube re-insertions by previous surgical procedures and age groups.**

	Recurrence of chronic OME			Test for failure time
Previous surgical procedures	n	%	P Value*	P Value†
**All age groups**				
First re-insertion				
Tube only (n = 988)	89	9.0	0.002	0.01
Tube+ adenoidectomy (n = 767)	39	5.1		
Second re-insertion				
Tube only (n = 68)	12	17.6	0.29	0.22
Tube+ adenoidectomy (n = 21)	1	4.8		
**Age stratification at first tube insertion**				
0–2 years				
Tube only (n = 178)	16	9.0	0.39	0.39
Tube+ adenoidectomy (n = 5)	1	20.0		
2–4 years				
Tube only (n = 143)	17	11.9	0.41	0.33
Tube+ adenoidectomy (n = 79)	13	16.5		
4–6 years				
Tube only (n = 422)	43	10.2	0.02	0.02
Tube+ adenoidectomy (n = 434)	25	5.8		
6–9 years				
Tube only (n = 245)	13	5.3	<0.001	<0.001
Tube+ adenoidectomy (n = 249)	0	0.0		

*Fisher's exact test was performed.

† Time to OME recurrence was tested by log-rank test.

Stratifying the children into four age groups (0–2 years, 2–4 years, 4–6 years, and 6–9 years), those older than 4 years old who received both adenoidectomy and tympanostomy tubes had statistically significant lower tube re-insertion rate and longer time to tube re-insertions than those who had tympanostomy tubes alone (Table 2 & Figure 2). (*p* = 0.02, *p*<0.001 for age group 4–6 and 6–9 respectively) There was no difference in tube re-insertions regardless of adenoidectomy in the age group 0–2 and 2–4 years (Table 2).

After controlling for age, adenoidectomy reduced the rate of tube re-insertion by 40% compared to tympanostomy tubes alone (aHR: 0.60; 95% CI: 0.41–0.89). After controlling for the effect of adenoidectomy, children who had their first tube surgery at the age of 2–4 years were most prone to tube re-insertions, followed by the 4–6 years age group (Table 3). Among 767 patients who received adenoidectomy, only two had severe post-operative bleeding that required intra-operative monitoring.

**Table 3:** **Estimated hazard rations (HR) and 95% confidence intervals (95% CI) of tympanostomy tube re-insertions of 2000–2001 birth cohort of chronic OME who had undergone tympanostomy tubes before 9 years of age.**

	Recurrence of chronic OME			
Variables	HR†	95% CI	aHR†	95% CI
Previous operation				
Tube only	1.00		1.00	
Tube+ adenoidectomy*	0.61	0.42–0.89*	0.60	0.41–0.89*
Age				
0–2 years	0.63	0.34–1.14	0.55	0.30–1.00*
2–4 years	1.00		1.00	
4–6 years	0.66	0.43–1.02	0.71	0.46–1.11
6–9 years	0.41	0.21–0.79*	0.44	0.23–0.86*

*p<0.05.

† HR = Hazard ratio; aHR = Adjusted hazard ratio; 95% CI = 95% confidence interval.

## Discussion

The 2000 and 2001 birth cohort in Taiwan had 565,666 children. Among them, 2221 had tympanostomy tube insertion before the age of 8 or 9 years for a cumulative incidence of 0.393%. Compared to other reports, one study showed the tympanostomy tube insertion rate in United states was 6.8% before the age of 3 and another study revealed middle ear surgical procedure was 9% in Norway [41], [42]. The rate of tube re-insertion is about 20% to 50% [8]–[10], [43]. The rate of tympanostomy tube insertion and tube re-sinsertion of children in Taiwan is low. This may be because Asian parents usually do not like their children to undergo surgery, leading to more conservative management or otolarygologists in Taiwan managed pediatric otitis media more conservatively under the suggestions of clinical practice guideline in comparison to surgeons in the United States [44]–[46].

This study demonstrates that adenoidectomy has a protective effect of preventing tube re-insertion in conjunction with the first tympanostomy tube insertion in children older than 4 years old compared to tube insertion alone. There were 849 cases in the 4–6 year old age group, which accounted for nearly half of the enrolled cases. Further stratifying this group into two groups of 4–5 years and 5–6 years for analysis, adenoidectomy had significant protective effects in the 4–5 year old age group but not in the 5–6 year old age group. The recurrence rate of children receiving adenoidectomy in the two age groups was 5.8% and 5.5%, respectively. The recurrence rates in tube only group was lower in the 5–6 year old age group (8.1%) than that in the 4–5 year old age group (12.1%). This may be due to the protective effect of age influencing the protective effect of adenoidectomy. We did not found the protective effect of adenoidectomy for children under 4 years old. Given small sample size for children under age of 4, post hoc power was calculated to examine whether the statistical power was large enough to detect differences in tube re-insertion rate between two surgical procedures. With an overall sample size of 183 0–2 years-old and 224 2–4 years-old children, the power achieves 37.1% and 33.6%, respectively, at a 0.05 significance level. This meant that there might be a protective effect which we could not detect due to small sample size for children under 4 years old.

After adjusting for the effect of age, adenoidectomy reduced the rate of tube re-insertion by 39%. These results are similar to those of most previous studies on this topic, most of them around 40% to 50% [10], [15], [17]–[19], [35]. If a child requires tube insertion at the age of 2–4 years, he or she are more likely to have tube re-insertions. This may be due to children in this age group are more likely to have recurrent AOM episodes, attending day care services, or shorter tubes staying time. Clinicians should therefore pay more attention to this age group of patients with chronic OME because they are prone to have recurrence. On the other hand, adenoidectomy is not beneficial to patients in this age group. Education the parents to avoid exposure to risk factors [46], medical management of allergic rhinitis, and vaccination for pneumococcal conjugate vaccine [47]–[49] are efforts that can be done in order to prevent the need for repeated tubes.

This study is the first to explore the problem using a population-based birth cohort. Every case born in the 2000 and 2001 were demonstrated and followed-up in this study without sampling to show what really happened to all these children in Taiwan who needed tympanostomy tube insertion before the age of 8 or 9 years. With the advantage of a population-based administrative database and the uniqueness a birth-cohort design, the numbers of tube insertions after birth of every case can be clearly defined and the concurrent surgical procedure (adenoidectomy or adeno-tonsillectomy) can be identified accurately without ambiguity in history.

To improve the internal validity of this study, tympanostomy tube insertion is used instead of diagnosis codes in ICD-9 as a surrogate of chronic OME and recurrent AOM for the accuracy of defining the study population. If there was a code for certain surgical procedures for a patient in the claims data, that patient definitely had the disease and underwent the surgical procedure for it on the date of the surgery. In contrast, if diagnosis codes in ICD-9 were used as a surrogate for the disease, the probability of miscoding by the physician might be much higher. Physicians might use a certain diagnosis code by misdiagnosis. They also might do this for prescribing antibiotics or laboratory test in order to pass the review of the insurance payer or to improve reimbursement.

The major limitation of this study is the limitation of the administrative claims data. Medical records and the operative notes of every patient could not be obtained. In the NHIRD, there was no clinical data like patient history, physical examination findings, laboratory data results, hearing level or surgical findings. Medical records could not be checked to identify if the patient had adenoid hypertrophy, adenitis, obstructive sleep apnea, or persistent purulent nasal discharge. The appearance of ear drum and culture results were also not known, which might lead to selection bias because surgeons perform adenoidectomy for more severe cases. Disease severity in the adenoidectomy group might be higher than in the tube insertion alone group. In the real world, a population based randomized control trial for this problem is not feasible or ethical. This study does offer an alternative way to explore the protective effects of adenoidectomy on tympanostomy tube re-insertions without any ethical issue. Other unobserved confounders are very likely to be diluted in this population based birth cohort study design and may have little influence.

Although adenoidectomy has protective effects on preventing tube re-insertions for children who need tympanostomy tubes, especially those older than 4 years old, performing adenoidectomy for every kid who needs tubes is not being recommended. The complication rate may not be high but there are complications due to the general anesthesia or from the procedure itself, including post-operative bleeding and nasopharyngeal stenosis [32]–[34]. Surgeons should take consider both the benefits and harm for every individual patient and make the best decision accordingly.

## Conclusions

Adenoidectomy has protective effect against the need for repeated tympanostomy tubes, especially for children older than 4 years. Children who need their first tube at the age of 2–4 years are most likely to have a tube re-insertion in the future. Surgeons should weigh the pros and cons for every individual patient before suggesting adenoidectomy to prevent recurrent chronic OME and AOM.
